# Prevalence and Distribution of Taurodontism and Pyramidal Molar Morphology in the Primary and Permanent Dentitions: A Retrospective Study in a Turkish Pediatric Population from Southeastern Anatolia

**DOI:** 10.3390/jcm15176876

**Published:** 2026-09-05

**Authors:** Hasan Said Şener, Emin Caner Tümen

**Affiliations:** Department of Pediatric Dentistry, Faculty of Dentistry, Dicle University, Diyarbakır 21280, Türkiye; dtsaidsener@gmail.com

**Keywords:** pyramidal molar, taurodontism, primary dentition

## Abstract

**Background/Objectives**: To evaluate the prevalence and distribution of taurodontism and pyramidal molar morphology in primary and permanent dentitions of children aged 6–16 years in Diyarbakır, Türkiye. **Methods**: A retrospective analysis of 1167 digital panoramic radiographs was conducted. Taurodontism was diagnosed according to the Shifman and Chananel criteria, while pyramidal molar morphology was evaluated as an independent anomaly. Prevalence, distribution, and symmetry were analyzed using Pearson’s chi-square test, Mann–Whitney U test, and Kappa test. **Results**: The individual and tooth prevalence of taurodontism was 6.60% and 2.44%, respectively, with significantly higher frequencies in the permanent dentition, maxilla, and permanent second molars (*p* < 0.001). Hypotaurodontism was the predominant subtype (81.5%). Pyramidal molar morphology was identified in 1.54% of individuals and 0.83% of teeth, with a significant female predominance (*p* = 0.022). Both anomalies exhibited high rates of bilateral involvement (85.7% for taurodontism and 72.2% for pyramidal molars). Pyramidal morphology was identified in 24 primary molars (0.58%). Among cases with available parental panoramic radiographs, the identification of the same morphology in at least one parent suggests potential familial aggregation, although further large-scale prospective genetic studies are required to confirm hereditary inheritance models. **Conclusions**: Taurodontism and pyramidal molar morphology are clinically significant developmental root variations in children. The quantitative documentation of pyramidal morphology in primary dentition addresses an important gap in the literature. Early radiographic recognition is essential for treatment planning and may serve as a helpful clinical indicator that prompts further genetic or systemic evaluation in suspected syndromic cases.

## 1. Introduction

Dental anomalies are structural, numerical, or positional deviations that arise from the complex interactions of genetic, epigenetic, and environmental factors during tooth morphogenesis [1]. Among these anomalies, taurodontism is one of the most distinctive variations affecting root morphology. Radiographically, it is characterized by marked vertical enlargement of the pulp chamber, apical displacement of the pulp chamber floor, absence of cervical constriction at the cemento-enamel junction (CEJ), and apical displacement of the root bifurcation [2,3]. The anomaly was first reported by deTerra in 1903 and later described in pre-Neanderthal fossils from Krapina by Gorjanović-Kramberger in 1908 [4]. The term taurodontism, meaning “bull tooth,” was subsequently introduced by Sir Arthur Keith in 1913 because of the resemblance of these teeth to the molar morphology of cattle and other ruminants [5]. Although Keith regarded this condition as a primitive trait, recent anthropological analyses suggest that this morphology may provide an adaptive advantage against wear associated with a hard diet [5,6]. Beyond its evolutionary significance, taurodontism is increasingly recognized as an important phenotypic marker of several genetic disorders and has been reported with a markedly higher prevalence in individuals with genetic syndromes, particularly Down syndrome [7]. Specifically, the chromosomal imbalance in Trisomy 21 is suggested to exert an inhibitory effect on cell proliferation during early odontogenesis, resulting in a global deceleration of dental formation and increased morphological variations [8,9]. This genetic linkage is further supported by a recent comparative investigation in a Turkish pediatric cohort by Cihangir and Kizilci (2026), which demonstrated that taurodontism is significantly more common in children with Down syndrome than in healthy peers (36.1% vs. 19.7%) [9].

Although the etiology of taurodontism has not been fully elucidated, the most widely accepted theory is insufficient or delayed horizontal invagination of Hertwig’s epithelial root sheath (HERS) [10,11,12]. At the molecular level, disturbances in the epithelial–mesenchymal interactions regulated by signaling molecules such as Sonic Hedgehog (SHH), Distal-Less Homeobox 2 (DLX2), and Patched 2 (PTCH2) are thought to contribute to the development of this anomaly [13,14,15]. Furthermore, the increased frequency of this anomaly in X chromosome aneuploidies, particularly Klinefelter syndrome (47, XXY), suggests that an X chromosome–linked genetic dosage effect may play a role in root morphogenesis [16,17,18].

Pyramidal molar morphology, which lies within a developmental spectrum similar to that of taurodontism, is characterized by the complete fusion of the roots into a single conical root containing one wide root canal [19,20]. Some authors consider pyramidal molar morphology to represent the most severe form of taurodontism [19], whereas others regard it as an independent anomaly resulting from the complete failure of HERS invagination [21].

The prevalence of taurodontism and pyramidal molar morphology has been reported to vary widely among populations, ranging from 0.1% to 48% [20,22,23,24]. However, the majority of existing studies have focused on permanent teeth, and data on the prevalence of taurodontism in the primary dentition remain limited [25,26]. Reports of pyramidal root morphology in the primary dentition, which is considered a rare finding, consist predominantly of case reports [20,27,28,29].

Early recognition of morphological variations in primary molars is crucial for the success of endodontic and restorative treatments. In taurodont and pyramidal teeth, the enlarged and elongated pulp chamber may increase the risk of unexpected bleeding and furcal perforation during access cavity preparation [11,25,26]. Furthermore, the anatomical variations of these teeth may influence the selection of materials used for pulp therapy and the retention of stainless steel crowns, which are frequently used in primary molars with extensive loss of tooth structure [25,30,31]. Therefore, an accurate assessment of these morphological variations is clinically important for preventing iatrogenic complications and ensuring appropriate treatment planning.

The aim of this study was to determine the prevalence of taurodontism and pyramidal molar morphology in the primary and permanent molars of children and adolescents representing the population of Diyarbakır, Türkiye, using the Shifman and Chananel metric index. In addition, this study aimed to address an important gap in the literature by comprehensively evaluating the quantitative distribution of pyramidal molar morphology in primary dentition and the distribution of these morphological variations according to age, sex, and anatomical location.

## 2. Materials and Methods

### 2.1. Study Design and Ethical Approval

This retrospective cross-sectional study was conducted using digital panoramic radiographs of pediatric and adolescent patients archived in the Department of Pediatric Dentistry, Faculty of Dentistry, Dicle University. All radiographs were previously obtained for diagnostic and treatment purposes. The study protocol was approved by the Clinical Research Ethics Committee of Dicle University (Approval No. 2024-02; Date: 31 January 2024), and the study was conducted in accordance with the ethical principles of the Declaration of Helsinki. Retrospective evaluation of both pediatric patient records and their parents’ existing records in the institutional database was covered under this protocol. Signed informed consent allowing the retrospective scientific use of completely anonymized diagnostic records was obtained from all patients or their legal guardians at the time of their clinical admission. The study population consisted of individuals aged 6–16 years who attended the clinic between 1 January 2018 and 1 January 2023. Only patients with complete archived records and signed informed consent forms were included. To ensure confidentiality, all patient data were anonymized before analysis.

### 2.2. Sample Selection

Digital panoramic radiographs obtained for diagnostic and treatment planning purposes between 1 January 2018 and 1 January 2023, were retrospectively reviewed. Archived records of pediatric and adolescent patients aged 6–16 years with visible developing primary and/or permanent molars were evaluated for eligibility criteria. Among the 1200 archived digital panoramic radiographs initially screened, 33 patients were excluded from the study due to poor radiographic quality or artifacts (n = 18), fixed orthodontic appliances obscuring the molar pulp chambers (n = 9), or extensive bilateral crown destruction and ongoing root canal therapy on the primary/permanent molars (n = 6). Consequently, a final sample of 1167 patients who met all inclusion criteria was included in the analysis.

The inclusion criteria were as follows: age between 6 and 16 years at the time of panoramic radiography; presence of primary molars and/or permanent first and second molars (excluding third molars) in the maxilla and/or mandible; teeth with completed apical foramen formation, except for pyramidal molars meeting the specific morphological criteria described below; availability of high-quality digital panoramic radiographs allowing adequate evaluation of the pulp chamber, root morphology, and clear visualization of the cementoenamel junction (CEJ); and absence of image artifacts or distortions that could interfere with the diagnostic assessment.

The exclusion criteria were a history of maxillofacial trauma or surgery; congenital malformations, cysts, or osseous pathologies affecting normal anatomical structures; fixed orthodontic appliances (e.g., brackets and bands) or coronal restorations (e.g., stainless steel crowns and extensive restorations) that prevented evaluation of the pulp chamber; teeth with deep dentinal caries, advanced coronal destruction, or previous endodontic treatment; teeth exhibiting advanced physiological or pathological root resorption that precluded metric measurements; unerupted teeth or molars with incomplete root development, except for pyramidal molars in which the characteristic solitary conical or cylindrical root configuration without furcation was already clearly identifiable despite incomplete apical closure; and radiographs with significant motion or metal artifacts.

### 2.3. Sample Size Calculation

The sample size of this study was determined based on the population of 492,764 children and adolescents aged 6–16 years residing in Diyarbakır, according to the 2022 data of the Turkish Statistical Institute (TurkStat, Ankara, Turkey). The minimum required sample size was calculated using the Raosoft Sample Size Calculator (Raosoft, Inc., Seattle, WA, USA). Based on a 99% confidence level, an assumed prevalence (response distribution) of 50%, and a 5% margin of error, the minimum required sample size was determined to be 663 participants. To enhance the reliability of the findings, 1167 patients who met the inclusion criteria were included in the analysis, substantially exceeding the calculated minimum sample size. Although this larger cohort enhances the statistical reliability of our data, the single-center and retrospective nature of the study requires caution when generalizing these findings to the broader population.

### 2.4. Classification of Study Groups

Specifically for the evaluation of taurodontism, to evaluate the developmental stages of the dentition accurately, the study population was categorized into three age groups: 6–7 years (representing primary dentition focus), 8–12 years (mixed dentition), and 13–16 years (representing the transition to permanent dentition and completion of root development in second molars).

In the 6–7 years age group, permanent first molars were excluded from the analysis because of incomplete root development (open apices), which precludes accurate and standardized metric measurements according to the Shifman and Chananel index; therefore, only primary molars were evaluated in this category. The 8–12 years group included both primary and permanent molars as part of the mixed dentition assessment, provided that the teeth met the inclusion criteria of completed apexogenesis. Patients in the 13–16 years group were evaluated primarily for permanent first and second molars, given that primary teeth had exfoliated and root development of second molars was sufficient for reliable metric analysis.

### 2.5. Panoramic Imaging Protocol

All digital panoramic radiographs included in the study were acquired using a Planmeca ProMax 2D panoramic imaging unit (Planmeca Oy, Helsinki, Finland) and processed using the Planmeca Romexis software suite version 2.9.2.R (Planmeca Oy, Helsinki, Finland). The imaging parameters were standardized at 70 kVp and 10 mA in the standard panoramic mode, with an exposure time of 15.9 s. The system was equipped with a 0.5 mm focal spot and 3.2 mm filtration.

### 2.6. Standardization of Images and Measurement Procedure

All digital panoramic radiographs included in the study were evaluated using the METASOFT Dentpacs PACS software version 2.112 (Metasoft Information Technologies, Eskişehir, Türkiye). The diagnosis and classification of taurodontism were performed according to the metric criteria described by Shifman and Chananel (1978) [32]. To ensure high measurement accuracy and overcome the limitations of 2D imaging in defining the cementoenamel junction (CEJ), the examiner utilized the system’s digital image enhancement tools, including high-magnification zoom, local contrast adjustment, and gray-scale inversion (negative mode), to clearly visualize the CEJ line before taking linear measurements. Radiographs where the CEJ could not be clearly and reliably identified due to distortion or superimposition were strictly excluded from the analysis.

**Figure 1:**
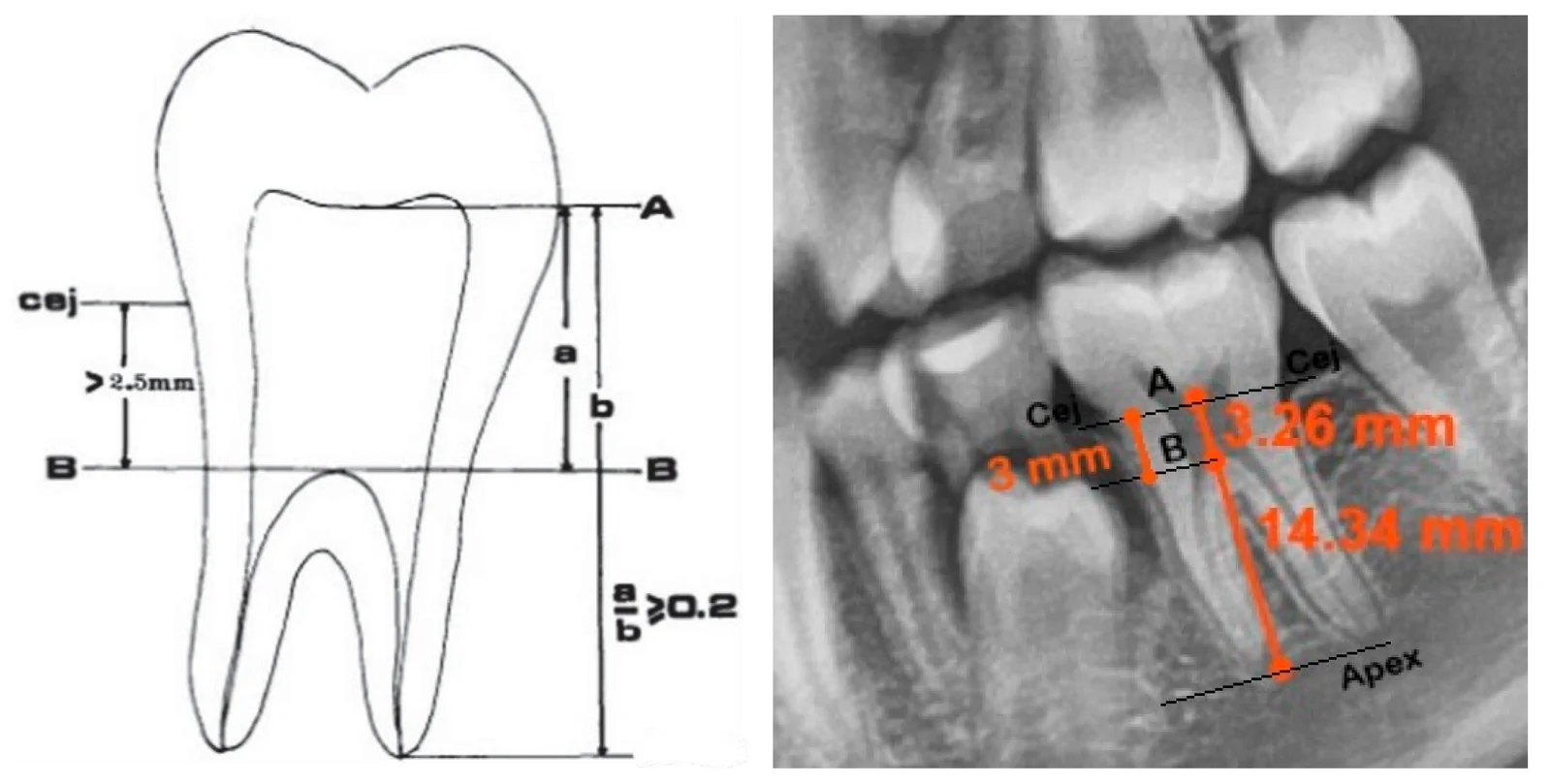
Shifman and Chananel classification system. (**Left**) Schematic diagram illustrating the anatomical reference points and variables used for measurements: lowest point of the pulp chamber roof (point A), highest point of the pulp chamber floor (point B), and cementoenamel junction (CEJ) line. (**Right**) Enlarged digital panoramic radiograph in the METASOFT Dentpacs software showing actual linear measurements of these reference points on a clinical case.

Three linear measurements were obtained from each molar tooth that met the inclusion criteria. The first measurement was defined as the vertical distance between the lowest point of the roof of the pulp chamber (A) and the highest point of the floor of the pulp chamber (B) (variable a). The second measurement was recorded as the vertical distance between the lowest point of the roof of the pulp chamber (A) and the apex of the longest root (variable b). The third measurement was defined as the vertical distance between the cemento-enamel junction (CEJ) and the highest point of the floor of the pulp chamber (B) (Figure 1).

The diagnosis of taurodontism was established when the Taurodont Index (TI = (a/b) × 100) was ≥20% and the third measurement exceeded 2.5 mm. According to the calculated Taurodont Index, teeth were classified as hypotaurodont (20.0–29.9%), mesotaurodont (30.0–39.9%), or hypertaurodont (40.0–75.0%). In addition, based on the third measurement, defined as the distance between the cemento-enamel junction and the floor of the pulp chamber, teeth were classified as hypotaurodont (2.5–3.7 mm), mesotaurodont (3.7–5.0 mm), or hypertaurodont (5.0–10.0 mm), and all measurements were recorded.

Pyramidal molar morphology was radiographically diagnosed based on the following specific criteria: (1) complete fusion of all roots into a single, undivided, conical or cylindrical root structure extending from the cementoenamel junction (CEJ) to the apex, with complete absence of root bifurcation or trifurcation (i.e., no furcation area); and (2) the presence of a single, wide root canal within the fused root. To ensure a clear differential diagnosis, pyramidal molars were strictly distinguished from hypertaurodont molars. In hypertaurodont molars, distinct root bifurcation or trifurcation remains present, although displaced apically into the apical third of the root, with multiple separate root canals emerging from the apically displaced pulp chamber floor. In contrast, pyramidal molars exhibit complete root fusion, with a single wide root canal and no root separation.

Accordingly, the presence of a solitary conical or cylindrical root without furcation and containing a single wide root canal was considered the diagnostic criterion for pyramidal molar morphology. Slight residual apical patency did not preclude classification when this characteristic root configuration was clearly identifiable radiographically. This approach is consistent with the methodology of Kwon et al., who included first and second molars at Demirjian developmental stages G and H in their radiographic assessment of pyramidal molar morphology. As stage G represents advanced root development with incomplete apical closure, complete apical closure was not considered a prerequisite for morphological assessment in their methodology [33].

### 2.7. Reliability Analysis

Intra-observer reliability was assessed using Cohen’s kappa coefficient to evaluate measurement reproducibility. To determine the consistency of the radiographic assessments, the selected images were re-evaluated by the same investigator two weeks after the initial assessment. The analysis revealed that the kappa values for all variables ranged from 0.854 to 1.000 and were statistically significant (*p* < 0.001). These findings indicate excellent intra-observer agreement and demonstrate that the measurements performed in this study were reproducible and reliable.

### 2.8. Statistical Analysis

Statistical analyses were performed using IBM SPSS Statistics for Windows, version 23.0 (IBM Corp., Armonk, NY, USA). Descriptive statistics were expressed as mean ± standard deviation and median (minimum–maximum) for continuous variables and as frequencies and percentages for categorical variables. The normality of data distribution was assessed using the Kolmogorov–Smirnov test.

Associations between categorical variables were analyzed using Pearson’s chi-square test, the chi-square test with Yates’ continuity correction, or Fisher’s exact test (including Monte Carlo simulation), as appropriate. Post hoc pairwise comparisons were performed using the Bonferroni-adjusted Z test. The Mann–Whitney U test was used to compare non-normally distributed continuous variables between two independent groups. Symmetry between the right and left sides (paired distribution) was evaluated using the McNemar test. Intra-observer agreement for the radiographic assessments was determined using Cohen’s kappa coefficient. A two-sided *p* value of <0.05 was considered statistically significant for all analyses.

## 3. Results

### 3.1. Participant Characteristics

A total of 1167 digital panoramic radiographs obtained from pediatric and adolescent individuals was included in this study. The participants ranged in age from 6 to 16 years, with a mean age of 10.62 ± 3.01 years. The study population consisted of 605 (51.8%) males and 562 (48.2%) females, indicating a balanced sex distribution. All participants were evaluated for taurodontism according to the Shifman and Chananel criteria and for pyramidal molar morphology according to the specific morphological criteria described above.

### 3.2. Findings Related to Taurodontism

#### 3.2.1. Overall Prevalence and Demographic Distribution

Taurodontism was identified in 77 patients, corresponding to an overall patient-based prevalence of 6.6%. According to sex, anomalies were detected in 41 males (6.8%) and 36 females (6.4%). Statistical analysis revealed no significant association between sex and the presence of taurodontism (*p* = 0.815), indicating that the condition occurred independently of sex in the study population.

A statistically significant association was observed between age group and the presence of taurodontism (*p* = 0.017). The highest prevalence was observed in the 13–16 years age group (9.5%), compared with the 8–12 years (5.2%) and 6–7 years (5.0%) age groups. The higher prevalence observed in the oldest age group may be attributed to the completion of root development in permanent second molars, allowing for a more reliable assessment of taurodontic morphology. Furthermore, when age was analyzed as a continuous variable, the mean age of patients with taurodontism was higher than that of patients without the anomaly (11.14 ± 3.01 vs. 10.58 ± 3.01 years); however, this difference did not reach statistical significance (*p* = 0.118, Mann–Whitney U test). The distribution of taurodontism across demographic variables is summarized in Table 1.

#### 3.2.2. Distribution by Dentition and Jaw Localization

A total of 9,085 molar teeth were evaluated, comprising 4971 permanent molars and 4,114 primary molars. Taurodontism was identified in 172 (3.46%) permanent teeth and 50 (1.22%) primary teeth. Statistical analysis demonstrated that the prevalence of taurodontism was significantly higher in permanent dentition than in primary dentition (*p* < 0.001).

Regarding jaw localization, taurodontic teeth were significantly more frequently observed in the maxilla (3.35%; n = 151) than in the mandible (1.55%; n = 71) (*p* < 0.001). This pattern was particularly evident in permanent dentition, where the frequency of taurodontism reached 4.76% (n = 120 out of 2521) in the maxilla compared with 2.12% (n = 52 out of 2450) (*p* < 0.001). In primary dentition, although a higher frequency of taurodontic molars was detected in the maxilla (1.56%; n = 31 out of 1991) than in the mandible (0.89%; n = 19 out of 2123), this difference did not reach statistical significance (*p* = 0.073). The distribution and intra-localization frequency of taurodontism across dentitions and jaws are summarized in Table 2.

#### 3.2.3. Tooth-Specific Prevalence and Molar Type Analysis

Analysis according to molar type revealed that in permanent dentition, second molars (5.0%; n = 78) exhibited a significantly higher prevalence of taurodontism than first molars (2.7%; n = 94) (*p* < 0.001). In contrast, no statistically significant difference was observed between primary first molars (1.1%; n = 22) and primary second molars (1.3%; n = 28) in the primary dentition (*p* = 0.818).

Regarding individual tooth numbers, the highest prevalence of taurodontism was observed in the maxillary permanent second molars, namely teeth 27 (6.8%) and 17 (6.5%). These were followed by the maxillary permanent first molars (tooth 26: 4.1%; tooth 16: 3.8%) and the mandibular permanent second molar (tooth 47:3.8%). The lowest prevalence in the study population was recorded for tooth 84 (0.6%). The tooth-specific prevalence and distribution of taurodontism are shown in Table 3.

#### 3.2.4. Classification of Taurodontism Degrees

The degree of taurodontism was evaluated in 222 affected teeth according to the quantitative criteria of Shifman and Chananel. Hypotaurodontism was the most common subtype, accounting for 81.5% (n = 181) of all cases, followed by mesotaurodontism (14.9%; n = 33) and hypertaurodontism (3.6%; n = 8).

A statistically significant difference was observed in the distribution of taurodontism subtypes between permanent and primary dentitions (*p* < 0.001). In the permanent dentition, hypotaurodontism, mesotaurodontism, and hypertaurodontism accounted for 77.9% (n = 134), 19.2% (n = 33), and 2.9% (n = 5), respectively. Mesotaurodontism was observed only in permanent dentition. In primary dentition, hypotaurodontism represented 94.0% (n = 47) of cases, whereas hypertaurodontism accounted for 6.0% (n = 3).

With respect to jaw localization, a significant difference in subtype distribution was observed between the maxilla and mandible in permanent dentition (*p* = 0.018). Hypotaurodontism was significantly more frequent in the mandible (90.4%) than in the maxilla (72.5%), whereas mesotaurodontism was significantly more frequent in the maxilla (24.2%) than in the mandible (7.7%). No significant difference was observed between the jaws in primary dentition (*p* = 1.000). The distribution of taurodontism subtypes according to dentition and jaw localization is summarized in Table 4.

#### 3.2.5. Distribution of Taurodontism Subtypes According to Age Group

Age-based analysis of the 222 affected teeth demonstrated a significant difference in the taurodontism subtype distribution among the age groups (*p* < 0.001). Hypotaurodontism remained the predominant subtype in all age groups; however, its frequency decreased from 93.3% in the 6–7 years age group to 70.1% in the 13–16 years age group. In contrast, mesotaurodontism increased progressively with age, reaching 29.9% in the 13–16 years age group. Hypertaurodontism was not observed in the oldest age group. The distribution of taurodontism subtypes according to age group is shown in Table 5.

#### 3.2.6. Relationship Between Taurodontism Subtypes and Molar Type

In permanent dentition, no significant association was observed between the taurodontism subtype and molar type in the mandible (*p* = 0.114). However, a significant association was identified in the maxilla (*p* = 0.040), where mesotaurodontism was more frequently observed in second molars (33.3%) than in first molars (17.4%).

When both jaws were evaluated together, a statistically significant relationship was found between taurodontism subtype and molar type in permanent dentition (*p* = 0.009). Hypotaurodontism was the predominant subtype in both first and second molars; however, mesotaurodontism was significantly more frequent in second molars (26.9%) than in first molars (12.8%). In contrast, hypertaurodontism was observed only in first molars (5.3%) and was not detected in second molars.

In the primary dentition, no significant association was observed between the taurodontism subtype and molar type in the mandible (*p* = 0.474), maxilla (*p* = 1.000), or overall analysis (*p* = 0.576). Hypotaurodontism was the predominant subtype in both primary first and second molars. The distribution of taurodontism subtypes according to the molar type is summarized in Table 6.

#### 3.2.7. Symmetry Analysis and Associated Dental Anomalies

The occurrence of taurodontism was evaluated with respect to symmetry and its association with other dental anomalies. Among the 77 patients diagnosed with taurodontism, 66 (85.7%) exhibited bilateral involvement and 11 (14.3%) showed unilateral involvement. Unilateral cases were similarly distributed between the right (n = 5; 6.5%) and left (n = 6; 7.8%) sides. McNemar test analysis revealed no significant difference between the right and left sides in the overall study population, permanent dentition, or primary dentition (*p* = 1.000 for all comparisons).

**Figure 2:**
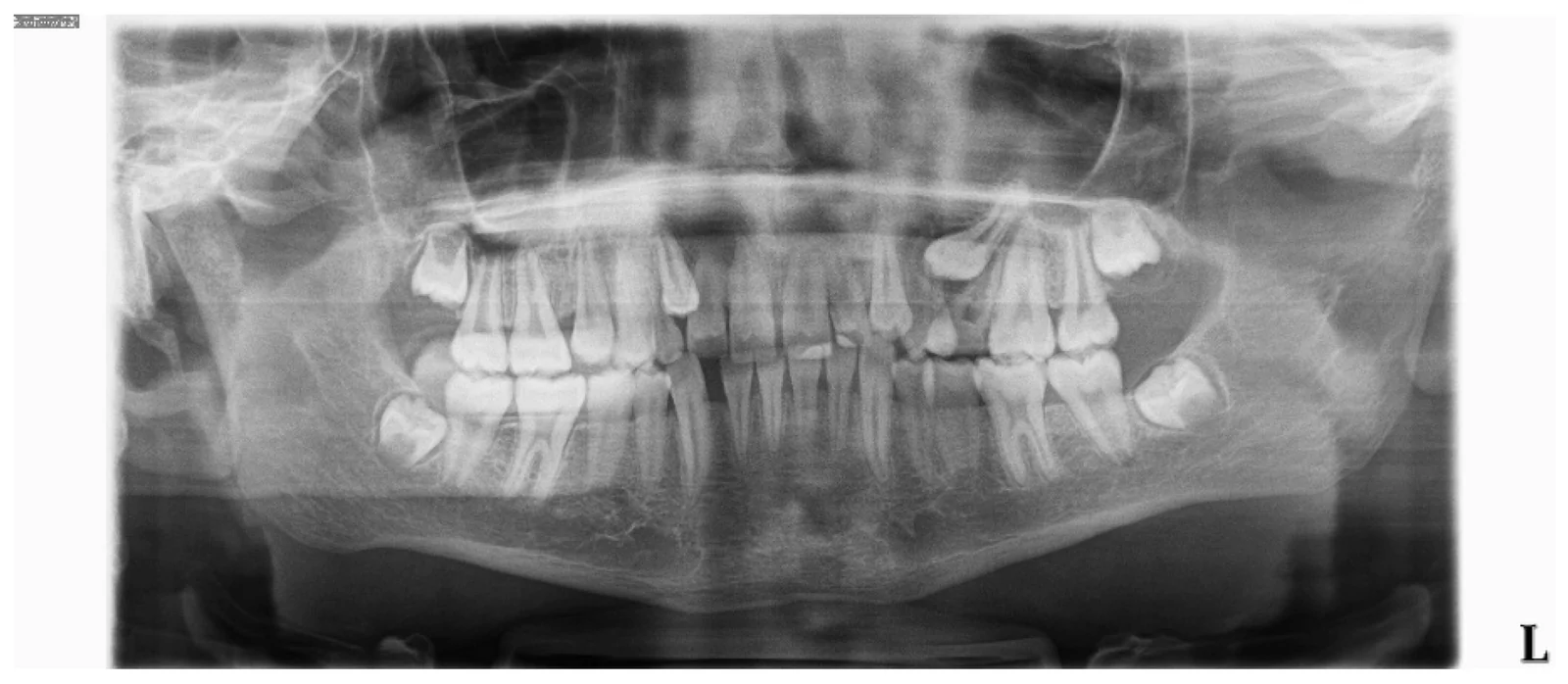
A panoramic radiograph from a 15-year-old girl demonstrating the coexistence of hypotaurodontism in the maxillary permanent first molars and pyramidal molar morphology in all permanent second molars.

With regard to associated dental anomalies, taurodontism occurred as an isolated finding in 62 patients (80.5%). Fifteen patients (19.5%) presented with at least one additional dental anomaly. Root dilaceration was the most frequently observed associated anomaly (10.4%; n = 8), followed by hypodontia (5.2%; n = 4). Pyramidal molar morphology was identified in 2.6% (n = 2) of patients (Figure 2), whereas pulp stones were detected in 1.3% (n = 1). The symmetry patterns and distribution of associated dental anomalies are presented in Table 7.

### 3.3. Findings Related to Pyramidal Molar Morphology

#### 3.3.1. Overall Prevalence and Demographic Distribution

Pyramidal molar morphology was identified in 18 of 1167 patients, corresponding to an overall patient-based prevalence of 1.54%. Regarding demographic variables, a statistically significant association was found between sex and the presence of pyramidal molars (*p* = 0.022). The anomaly was detected in 14 females (2.49%) and 4 males (0.66%). In terms of continuous age distribution, no statistically significant difference was found between the patients with pyramidal molar morphology and the unaffected group (*p* > 0.05, Mann–Whitney U test).

Tooth-based analysis was performed on 9085 molars. Pyramidal morphology was identified in 75 teeth, corresponding to an overall tooth-based prevalence of 0.83%. The prevalence was significantly higher in permanent teeth (1.03%; n = 51) than in primary teeth (0.58%; n = 24) (*p* = 0.025). Among the 18 affected patients, 15 presented the anomaly in the permanent dentition, whereas 3 presented the anomaly in the primary dentition. The distribution of pyramidal molars according to demographic and dentition-based variables is summarized in Table 8.

#### 3.3.2. Analysis by Molar Type (First vs. Second Molars)

A tooth-based analysis was conducted to determine the distribution of pyramidal morphology between the first and second molars. In permanent dentition, the anomaly was significantly more prevalent in second molars (2.19%; n = 34) than in first molars (0.50%; n = 17) (*p* = 0.001).

In the primary dentition, pyramidal morphology was identified in 12 primary first molars (0.62%) and 12 primary second molars (0.55%), with no statistically significant difference between the two molar types (*p* > 0.05). The distribution of pyramidal molars according to the molar type is presented in Table 9.

#### 3.3.3. Jaw Localization and Side Distribution

The localization of the 75 pyramidal molars was evaluated according to the jaw and side distribution. No significant association was found between the presence of pyramidal molars and jaw localization (*p* = 0.819). Overall, 39 teeth (52.0%) were located in the maxilla and 36 teeth (48.0%) were located in the mandible.

The analysis of side distribution also revealed no significant difference between the right and left sides (*p* = 0.733). Pyramidal molars were distributed almost equally between the right (50.7%; n = 38) and left (49.3%; n = 37) sides of the dental arches. The distribution of pyramidal molars according to jaw localization and side is presented in Table 10.

#### 3.3.4. Symmetry Analysis

Patient-based symmetry analysis of 18 individuals with pyramidal molars revealed a predominance of bilateral involvement. Bilateral occurrence was observed in 13 patients (72.2%), whereas 5 patients (27.8%) exhibited unilateral involvement. In the primary dentition, all identified cases showed bilateral involvement.

McNemar test analysis revealed no significant difference between the right and left sides in the overall study population, permanent dentition, or primary dentition (*p* = 1.000 for all comparisons). The symmetry characteristics of pyramidal molars are presented in Table 11.

#### 3.3.5. Familial Distribution of Pyramidal Molar Morphology

**Figure 3:**
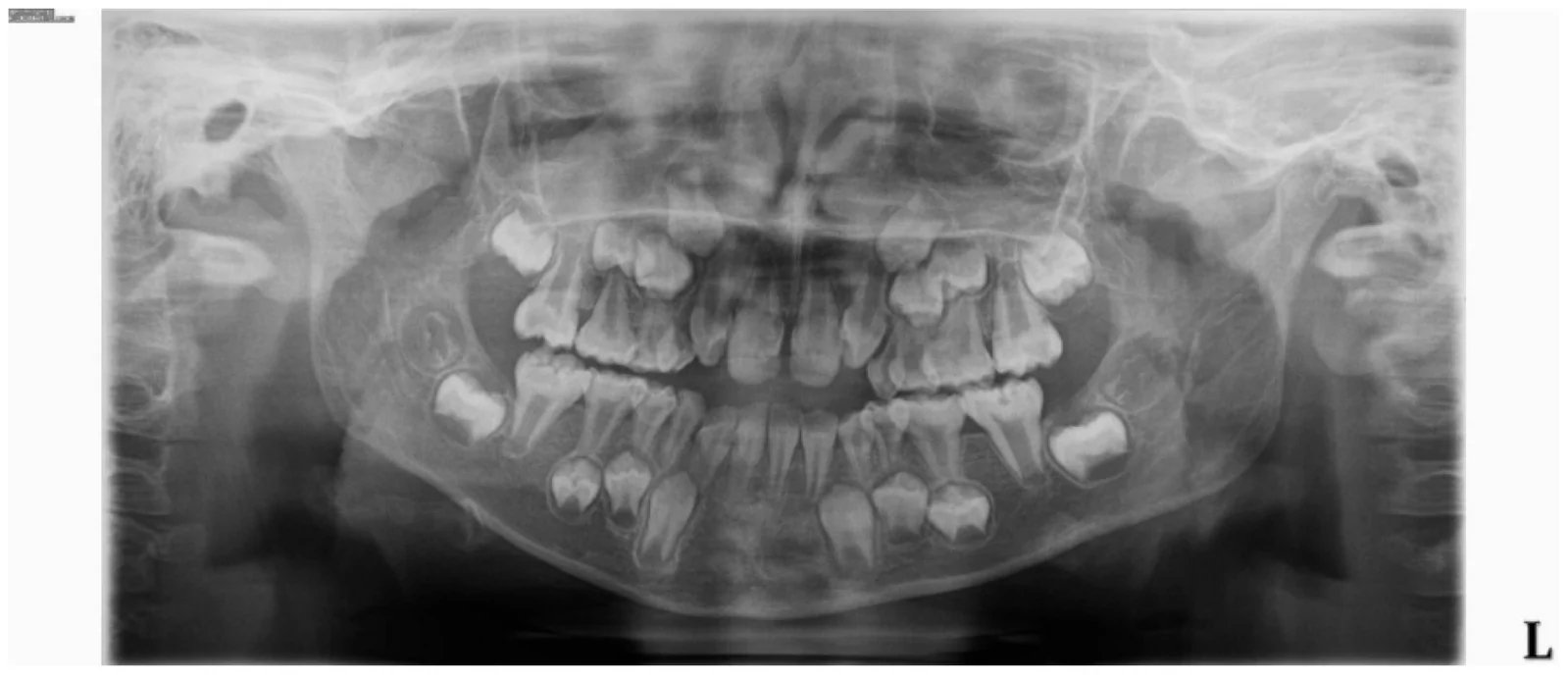
Panoramic radiograph of a 6-year-old girl included in the present study, demonstrating pyramidal molar morphology in both primary and permanent molars.

**Figure 4:**
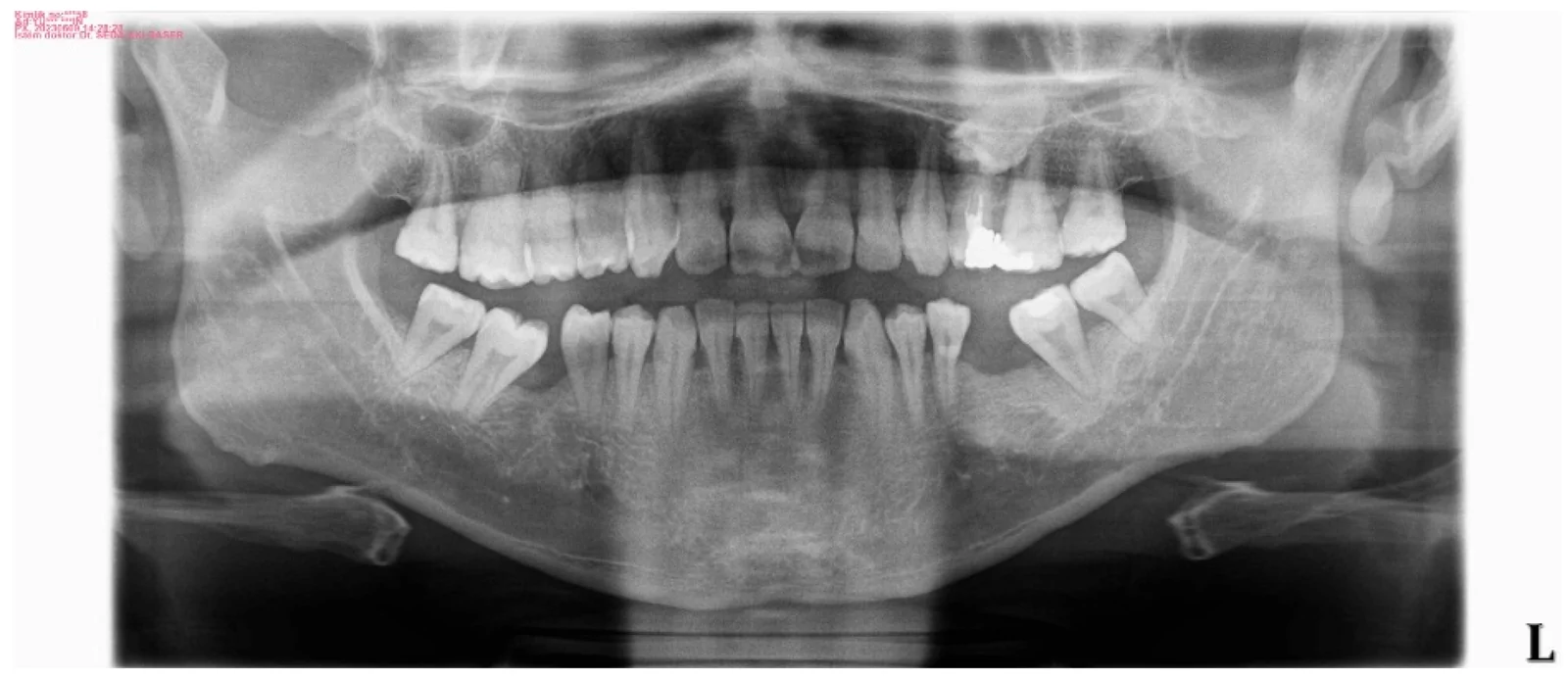
A panoramic radiograph of the mother of the patient is shown in Figure 3, demonstrating similar pyramidal molar morphology in the molar teeth.

**Figure 5:**
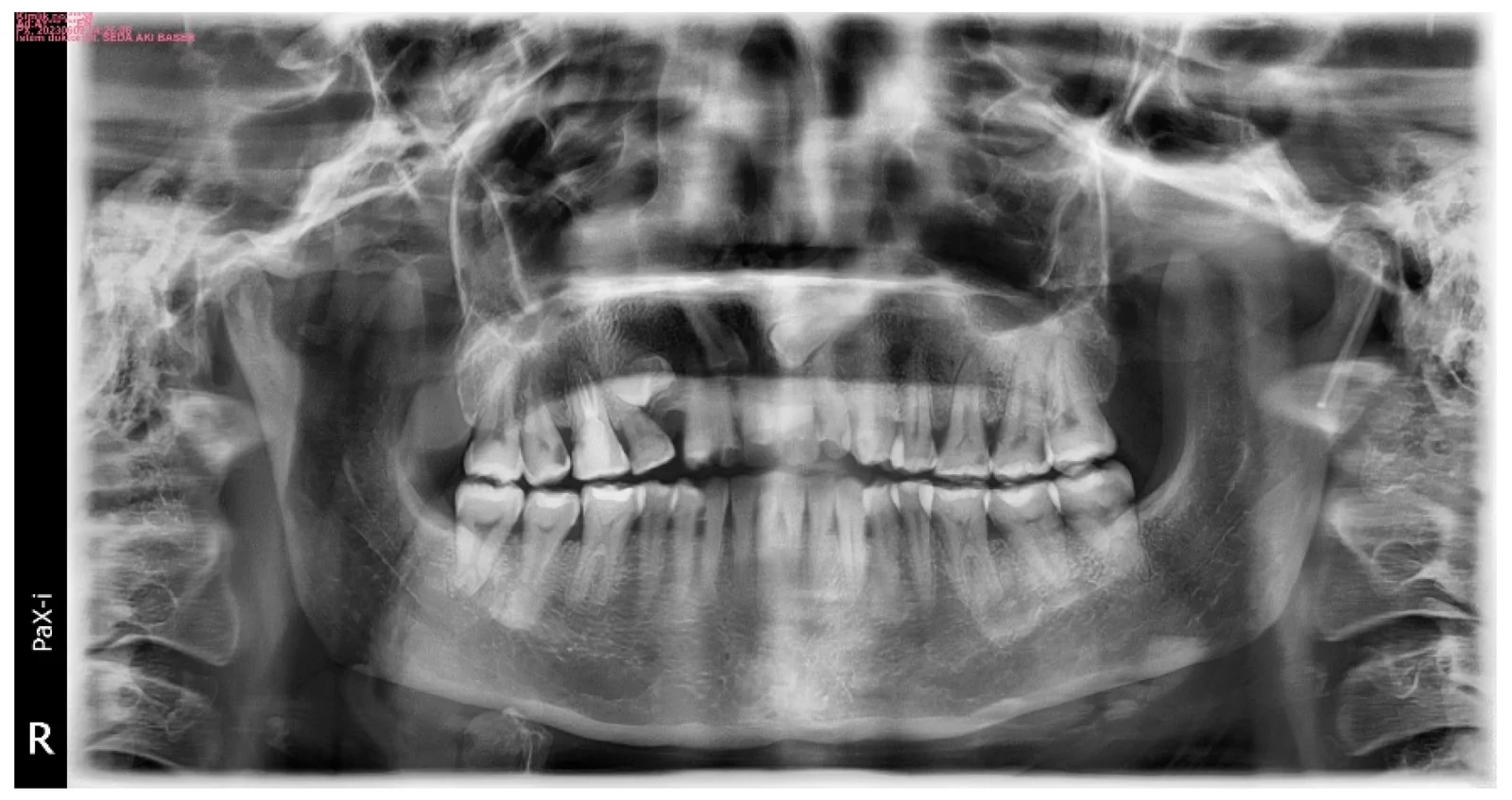
A panoramic radiograph of the father of the patient is shown in Figure 3, demonstrating similar pyramidal molar morphology in the molar teeth.

As an additional observation, parental panoramic radiographs available in the institutional radiographic archive were retrospectively reviewed for 10 of the 18 patients diagnosed with pyramidal molar morphology (Figure 3). These parents were not part of the original study population but had panoramic radiographs available in the institutional database. Pyramidal molar morphology was identified in at least one parent of all evaluated individuals. Among the three patients presenting with pyramidal molars in the primary dentition, both parents demonstrated pyramidal molar morphology. In the remaining seven patients with pyramidal molars in the permanent dentition, pyramidal molar morphology was observed in at least one parent (Figure 4 and Figure 5).

## 4. Discussion

In the present study, the individual-based prevalence of taurodontism was 6.60%, whereas the tooth-based prevalence was 2.44%. Taurodontism was found to be significantly more common in permanent dentition, maxillary molars, and second molars, with bilateral involvement observed in the majority of affected individuals. These findings provide comprehensive epidemiological data regarding the distribution patterns of taurodontism in a pediatric population from southeastern Türkiye.

The prevalence of taurodontism varies widely in the literature, from less than 0.1% to 48% [34,35], due to differences in demographics, diagnostic criteria, and imaging modalities. Our 6.6% prevalence is consistent with international reports using similar quantitative criteria, such as Shifman and Chananel (5.6% in Israel) [32] and Bronoosh et al. (5.5% in Iran) [6]. In pediatric cohorts, Jorgensen et al. reported a prevalence of 4.37% among African children [36], supporting a consistent distribution across younger populations when standardized metrics are applied.

Within Türkiye, reported prevalence rates vary from 0.02% to 22.8% [37,38]. The high rate (22.8%) reported by Topçuoğlu et al. is likely due to the inclusion of third molars, which are highly prone to morphological variations [38]. Since third molars were excluded here due to our pediatric cohort, our study provides a focused, age-specific estimate of taurodontism prevalence in children and adolescents.

The wide variation in prevalence rates reported across studies may partly be explained by differences in diagnostic methodology. In the present study, taurodontism was assessed using the metric criteria proposed by Shifman and Chananel [32], which provide a more objective and reproducible diagnostic approach than subjective visual evaluation. While visual assessment relies heavily on examiner interpretation, metric indices allow the identification of mild forms of taurodontism that might otherwise remain unrecognized. This may partly explain the remarkably low prevalence of 0.02% reported by Koparal et al. in an Adıyaman population, a geographically neighboring region to the present study area, where taurodontism was evaluated using a visual assessment method [37]. Furthermore, the predominance of hypotaurodontism in the present study, accounting for 81.5% of all taurodont teeth, supports the notion that mild forms of the anomaly are more readily identified when quantitative diagnostic criteria are employed. Therefore, methodological differences should be carefully considered when comparing prevalence rates across studies.

Consistent with the majority of previous epidemiological studies [25,32,39,40], no significant association was found between sex and the prevalence of taurodontism in the present study (6.8% in males vs. 6.4% in females; *p* = 0.815). Although some regional investigations have reported female predominance and suggested a potential influence of X-linked genetic factors [6,41,42], the current findings do not support sex-related predisposition. These results suggest that taurodontism is more likely to represent a developmental anatomical variation that occurs independently of sex in the pediatric population.

In contrast, a significant association was observed between age group and the presence of taurodontism (*p* = 0.017). The prevalence increased from 5.0% in the 6–7-year age group and 5.2% in the 8–12-year age group to 9.5% in the 13–16-year age group, indicating a marked increase in adolescence. However, this increase is more likely attributable to the completion of root development in permanent second molars by the age of 13–16 years, allowing reliable assessment according to the Shifman and Chananel criteria rather than reflecting a biological progression of the anomaly over time. Furthermore, according to Shaw’s molar field theory, susceptibility to taurodontism increases toward the posterior members of the molar series, with the second and third molars being the teeth most frequently affected by this anomaly [21]. Consistent with this theory, the prevalence of taurodontism was significantly higher in permanent second molars than in permanent first molars in the present study (5.0% vs. 2.7%; *p* < 0.001).

In pediatric cohorts aged 6–16 years, active root development and incomplete secondary dentin deposition naturally enlarge the pulp chamber, potentially causing false-positive taurodontism diagnoses. To address this, for the assessment of taurodontism, we strictly included only teeth with completely formed apices. Furthermore, using the Shifman and Chananel double-threshold criteria—requiring both a ratio (TI ≥ 20%) and an absolute vertical distance of >2.5 mm from the CEJ to the pulp floor—served as a crucial safeguard, preventing the misclassification of teeth with wide pulp chambers but normal pulp floor levels.

Permanent taurodont teeth were significantly more frequent in the maxilla (4.76%) than in the mandible (2.12%), indicating a marked maxillary predominance of the anomaly. This finding is consistent with a recent study by Pach et al. (2023), which reported that taurodontism predominantly affected maxillary teeth (52%) compared with mandibular teeth (16%) in a contemporary Polish population [4], as well as with the findings of Li et al. (2025), who observed a significant maxillary predominance (9.06% vs. 5.15%) in a Chinese population [43]. Similarly, several studies conducted in the Turkish population, including those by Eymirli et al. (2025) [39], Elbay et al. (2016) [44], and Çelikoğlu et al. (2010) [45], have also documented a higher frequency of taurodontism in the maxillary region. This pattern may be related to the more complex three-rooted anatomy and furcation development of maxillary molars, which could be more susceptible to disturbances affecting Hertwig’s epithelial root sheath (HERS) than the relatively simpler two-rooted mandibular molars. In addition, the greater anatomical complexity of the maxilla has been suggested as a possible factor contributing to its increased susceptibility to developmental disturbances.

Consistent with Shaw’s theory, as previously mentioned regarding the increased susceptibility of posterior molars [21], the highest prevalence rates in the current study were observed in the maxillary second molars, particularly teeth #27 (6.8%) and #17 (6.5%); however, this trend was not observed in the primary dentition, where the anomaly was distributed more uniformly between the primary first and second molars.

Although the literature has predominantly focused on permanent dentition, the present study also evaluated primary molars and identified a tooth-based prevalence of 1.22%. This finding is identical to that reported by Simsek et al. in a Turkish pediatric population [25], suggesting a relatively consistent prevalence pattern of taurodontism in primary molars across different regions of Türkiye. Although less frequent than in permanent molars (3.46%), the presence of taurodontism in primary dentition remains clinically relevant. The enlarged pulp chamber and apically displaced pulpal floor characteristic of taurodont teeth may increase the risk of pulp exposure during caries progression and complicate endodontic procedures by increasing the likelihood of furcal perforation and difficulties in canal identification and instrumentation [11,30,46].

In the present study, taurodontism exhibited a predominantly bilateral and symmetrical distribution, with bilateral involvement observed in 85.7% of affected individuals. This high degree of symmetry is consistent with previous reports [32,44,47,48,49] and suggests that taurodontic morphology is primarily a result of intrinsic developmental and genetic programs rather than localized environmental influences. With respect to severity, hypotaurodontism constituted the vast majority of cases (81.5%). In contrast, hypertaurodontism, the most severe form of anomaly, was rare (3.60%) and was identified predominantly in maxillary molars, with a few cases also observed in the mandible and primary dentition.

When the distribution of taurodontism subtypes was examined, the severity of the anomaly appeared to be associated with jaw location, age group, and molar type. In the permanent dentition, mesotaurodontism was observed more frequently in the maxilla than in the mandible (24.2% vs. 7.7%; *p* = 0.018), whereas hypotaurodontism accounted for the majority of cases in the mandible. Similarly, the increase in the frequency of mesotaurodontism and the corresponding decrease in hypotaurodontism in the 13–16-year age group (*p* < 0.001) may be related to the inclusion of permanent second molars after the completion of root development. Furthermore, mesotaurodontism was observed approximately twice as often in the second molars as in the first molars (26.9% vs. 12.8%; *p* = 0.009). Taken together, these findings indicate that hypotaurodontism, the mildest form of the anomaly, predominates across all categories, whereas more pronounced morphological alterations tend to occur more frequently in the maxilla, during adolescence, and in the second molars.

The association of taurodontism with other dental anomalies has been extensively investigated [10,19,20,50]. In the present study, taurodontism was an isolated finding in the majority of affected individuals (80.5%). However, at least one additional dental anomaly was identified in 19.5% of cases, most commonly root dilaceration (10.4%) and hypodontia (5.2%). Although these findings suggest that taurodontism generally occurs as an independent morphological variation, its association with other developmental dental anomalies indicates that shared developmental pathways may occasionally be involved.

Root dilaceration was the most frequently associated anomaly in the present study. Given that both taurodontism and dilaceration involve alterations in root morphology and are linked to the development of HERS, their co-occurrence may reflect common disturbances during root development [11,51]. Similarly, hypodontia was identified in 5.2% of the affected individuals. As both hypodontia and taurodontism are developmental anomalies that originate during the early stages of odontogenesis, their association may be attributable to overlapping genetic and embryological mechanisms [10].

An additional finding of interest was the presence of pyramidal molars in a small number of taurodontic individuals. Since both pyramidal molars and taurodont teeth are characterized by abnormalities in root formation and furcation development, their coexistence further supports the concept that disturbances affecting HERS may contribute to a spectrum of root developmental anomalies [19,20]. Our findings regarding the co-occurrence of these anomalies are strongly supported by Ozgur et al. (2025), who identified a statistically significant association between the number of taurodontic teeth and the presence of pyramidal molars (*p* < 0.05) in a Turkish pediatric cohort aged 9–15 years, reinforcing the hypothesis of a shared etiopathogenic pathway involving HERS invagination failures [52]. Collectively, these findings suggest that although taurodontism most often presents as an isolated condition, its occasional association with other dental anomalies may reflect the common developmental processes underlying tooth morphogenesis.

Pyramidal molar morphology, characterized by the complete fusion of roots into a single conical structure with a wide canal, represents an extreme manifestation of developmental variations in HERS. According to the threshold inheritance model proposed by Ackerman et al., normal root morphology, taurodontism, and pyramidal molars exist along a continuous morphological spectrum [19,20]. Our findings support this concept, as pyramidal molars were identified as an independent but related anomaly, occurring in 1.54% of individuals and 0.83% of all examined teeth. The underlying etiology is attributed to the failure of HERS to invaginate and initiate furcation formation during early root development, a process regulated by several molecular signaling pathways, including Sonic Hedgehog (SHH) and Distal-Less Homeobox 2 (DLX2) [13,14,15].

The most significant contribution of the present study is the comprehensive documentation of the epidemiological characteristics of pyramidal morphology in primary molars, including its prevalence, sex distribution, maxillary and mandibular involvement, bilateral symmetry, and the types of teeth affected. The available literature on pyramidal molar morphology in primary dentition is limited to case reports [27,29,53,54]. Furthermore, while previous large-scale pediatric studies, such as those by Klein et al. (examining more than 10,000 children) and Eymirli et al. (2025), reported no cases of pyramidal morphology in primary molars [20,39], our study identified 24 primary molars (0.58%) exhibiting this anomaly, representing the largest pediatric cohort presented in the literature. The extreme rarity of pyramidal or monoradicular morphology in the primary dentition is further highlighted by the recent literature; Almulhim (2026) reported that monoradicular primary maxillary second molars represent an exceptionally rare developmental anomaly, with only six cases documented globally to date [55]. In this context, our identification of pyramidal molar morphology in 24 primary molars (0.58%) represents the largest pediatric cohort presented in the literature, providing valuable epidemiological reference data for early childhood. This finding fills an important epidemiological gap in the literature regarding the prevalence and distribution characteristics of pyramidal molar morphology in primary dentition. The presence of this variation in primary teeth further suggests that disturbances in HERS invagination may manifest during the earliest stages of tooth root development.

In the present study, pyramidal molar morphology was significantly more prevalent in females (2.49%) than in males (0.66%) (*p* = 0.022). However, given that pyramidal molar morphology was identified in only 18 of the 1,167 participants, these subgroup findings, including the sex-based comparison, should be interpreted with caution due to the limited statistical power and potential uncertainty associated with small sample sizes. This is consistent with Klein et al. (3.1-fold higher in females) and other Turkish studies reporting female predominance [20,47,56]. The localization of root morphogenesis genes on the X chromosome [15,40], supported by the higher frequency of root variations in Klinefelter syndrome, has been proposed as a genetic basis [16,57,58]. Interestingly, while taurodontism showed no sex predilection (*p* = 0.815), pyramidal molar morphology was significantly female-biased. This epidemiological divergence suggests that pyramidal molars might represent a distinct root developmental anomaly [2,13] with its own characteristics rather than merely an advanced stage of taurodontism [19,20], though further molecular confirmation is needed.

Another noteworthy finding of the present study was the pyramidal molar morphology observed in the parents of the affected individuals. The literature suggests that pyramidal molar morphology exhibits a strong familial predisposition and that root morphology is largely determined by genetic factors [54,58]. Nguyen et al. (1996) reported pyramidal molar morphology and canine-like morphological features in multiple members of the same family, highlighting the hereditary nature of this anomaly [54]. Similarly, Robbins and Keene (1964) proposed an autosomal dominant mode of inheritance after documenting the transmission of pyramidal root morphology through four successive generations without skipping a generation [59]. In contrast, Ackerman et al. (1973) interpreted the inheritance of pyramidal molar morphology within the framework of the threshold inheritance model and suggested that the anomaly could be explained by a polygenic mechanism involving the contribution of multiple genes [19].

In the present study, pyramidal molar morphology was identified in at least one parent of every affected individual for whom parental panoramic radiographs were available. Furthermore, both parents exhibited pyramidal molar morphology in all three patients presenting this anomaly in the primary dentition. This observation is consistent with the hypothesis of Ackerman et al. [19] that the most severe phenotypes may result from the cumulative effect of genetic load, as well as with the familial findings reported by Nguyen et al. [54]. Although these findings are based on a limited number of cases, they suggest that pyramidal molar morphology occurring in the primary dentition may be associated with a stronger hereditary component.

Nevertheless, this evaluation was based solely on a limited number of parents for whom panoramic radiographs were available and was not designed as a systematic familial investigation. In addition, because one or more molars were missing in several parents, pyramidal morphology could not be assessed in the extracted or absent teeth. Therefore, the present findings should be interpreted with caution and confirmed through larger family-based and genetic studies in the future.

Similar to taurodontism, pyramidal molar morphology in permanent dentition was significantly more prevalent in second molars (2.19%) than in first molars (0.50%) in the present study, consistent with previous reports (*p* = 0.001) [20,47,56]. This finding supports the concept that later-developing teeth within the molar series are more susceptible to disturbances in HERS invagination. In contrast, no significant difference was observed between primary first and second molars, suggesting that the developmental disturbance may be more uniformly distributed throughout the primary dentition.

Consistent with previous reports [20,24], the high rate of bilateral symmetry observed in the present study (72.2% overall and 100% in the primary dentition) suggests that pyramidal root formation is driven by systemic genetic determinants rather than being a consequence of localized external or traumatic events. Furthermore, the absence of a significant difference between the maxilla and mandible (*p* = 0.819) supports the hypothesis that, once the genetic threshold required for pyramidal molar development is reached, the anomaly may manifest similarly in both jaws.

The unique anatomical characteristics of teeth exhibiting taurodont and pyramidal molar morphologies present several clinical challenges and require special consideration during treatment planning [11,32]. From an endodontic perspective, the enlarged pulp chamber and apically displaced pulpal floor observed in taurodont teeth increase the risk of iatrogenic perforation, particularly during access cavity preparation [11,30,31]. In addition, the complex root canal anatomy of these teeth, together with the single, wide root canal characteristic of pyramidal molars, may necessitate the use of potent irrigants, such as 2.5% sodium hypochlorite, and modified obturation techniques to ensure the effective removal of necrotic tissue [60,61].

In restorative and orthodontic treatments, the absence of cervical constriction in primary teeth exhibiting pyramidal morphology may compromise the adaptation and long-term retention of stainless steel crowns (SSCs), particularly in pediatric patients [30,31]. Furthermore, the reduced root surface area associated with the conical and fused root configuration may provide less orthodontic anchorage, requiring clinicians to exercise greater caution to minimize the risk of anchorage loss [54,62].

In conclusion, early recognition of these morphological variations on panoramic radiographs is clinically important not only for optimizing treatment planning and preventing potential complications but also for facilitating the early identification of genetic syndromes associated with taurodontism [16,19,63].

This study had several limitations that should be considered when interpreting the findings. First, the exclusive use of panoramic radiography may have limited the detailed evaluation of root morphology because of the inherent limitations of panoramic imaging, including magnification, distortion, and anatomical superimposition. In addition, the routine use of cone-beam computed tomography (CBCT) is not feasible in the pediatric population because of radiation exposure and ethical considerations [64], thereby limiting a more precise assessment of taurodontism severity. Owing to the retrospective design of the study, systemic diseases, syndromic findings, family history, and genetic characteristics could not be comprehensively evaluated, and genetic analyses were not performed in this study. Furthermore, the exclusion of third molars and paramolars from the study, together with the use of only the Shifman and Chananel index for the diagnosis of taurodontism, represents an additional methodological limitation. Finally, the familial evaluation of pyramidal molar morphology was based only on a limited number of parents for whom panoramic radiographs were available and was not designed as a systematic family-based investigation. Additionally, the small number of individuals diagnosed with pyramidal molar morphology (n = 18) limits the statistical power of subgroup analyses, introducing some uncertainty that necessitates larger multicenter studies to confirm these trends. Therefore, further prospective studies incorporating three-dimensional imaging, genetic analyses, and larger family cohorts are needed to improve our understanding of the etiopathogenesis of taurodontism and the morphology of pyramidal molars.

## 5. Conclusions

This study provides comprehensive quantitative data on the prevalence and distribution of taurodontism and pyramidal molar morphology in a pediatric population from southeastern Türkiye. Taurodontism was more prevalent than pyramidal molar morphology, occurring predominantly in the permanent dentition, maxilla, and permanent second molars, with hypotaurodontism representing the most common subtype. The predominantly bilateral distribution observed for both anomalies provides strong evidence for a shared developmental origin, reinforcing the role of systemic genetic factors.

One of the major contributions of this study is the documentation of the prevalence and distribution characteristics of pyramidal molar morphology in primary dentition, providing novel reference data for this anomaly in early childhood. In addition to the marked female predominance observed for pyramidal molars, the identification of the same morphology in at least one parent of the patients for whom parental panoramic radiographs were available represents a noteworthy observation, suggesting that this anomaly may exhibit familial aggregation. However, these findings should be confirmed through larger family-based studies and comprehensive genetic investigations.

From a clinical perspective, early recognition of these morphological variations on routine panoramic radiographs is of great importance for appropriate treatment planning and prevention of iatrogenic complications during pediatric endodontic, restorative, and orthodontic procedures. Furthermore, the identification of these anomalies should raise clinical awareness regarding their documented associations with various developmental and genetic syndromes, highlighting the importance of multidisciplinary referral when clinically indicated.

Future multicenter prospective studies incorporating three-dimensional imaging, comprehensive genetic analyses, and systematic family-based investigations are needed to improve our understanding of the biological basis and potential hereditary mechanisms underlying taurodontism and pyramidal molar morphologies.

## Figures and Tables

**Table 1:** Demographic characteristics of the study population and distribution of taurodontism.

Variables	Total Patients (N = 1167)	Taurodontism Present (n = 77)	Taurodontism Absent (n = 1090)	*p*
Sex				0.815
Male	605 (51.8%)	41 (6.8%)	564 (93.2%)	
Female	562 (48.2%)	36 (6.4%)	526 (93.6%)	
Age Groups				0.017
6–7 years	220 (18.8%)	11 (5.0%)	209 (95.0%)	
8–12 years	559 (47.9%)	29 (5.2%)	530 (94.8%)	
13–16 years	388 (33.3%)	37 (9.5%)	351 (90.5%)	
Total	1167 (100%)	77 (6.6%)	1090 (93.4%)	

Pearson’s chi-square test was used for statistical comparisons. Statistical significance was set at *p* < 0.05.

**Table 2:** Distribution and intra-localization frequency of taurodontism according to dentition type and jaw localization.

Dentition	Taurodontism	Mandible n (%)	Maxilla n (%)	Total n (%)	*p*
Permanent	Normal	2398 (97.88%)	2401 (95.24%)	4799 (96.54%)	<0.001 ^a^
	Taurodont	52 (2.12%)	120 (4.76%)	172 (3.46%)	
Primary	Normal	2104 (99.11%)	1960 (98.44%)	4064 (98.78%)	0.073 ^b^
	Taurodont	19 (0.89%)	31 (1.56%)	50 (1.22%)	
Total	Normal	4502 (98.45%)	4361 (96.65%)	8863 (97.56%)	<0.001 ^a^
	Taurodont	71 (1.55%)	151 (3.35%)	222 (2.44%)	

^a^ Pearson’s chi-square test; ^b^ Yates’ continuity correction. Values are presented as n (%). Percentages were calculated within each jaw and dentition category (column percentages). Statistical significance was set at *p* < 0.05.

**Table 3:** Prevalence and distribution of taurodontism according to tooth number and molar type.

Dentition	Molar Type	Tooth Number	Evaluated (N)	Taurodont (n)	Prevalence (%)	*p*
Permanent	First Molar	Total	3419	94	2.7 ^a^	<0.001 ^x^
		16	876	33	3.8	
		26	878	36	4.1	
		36	819	13	1.6	
		46	846	12	1.4	
	Second Molar	Total	1552	78	5.0 ^b^	
		17	384	25	6.5	
		27	383	26	6.8	
		37	392	12	3.1	
		47	393	15	3.8	
Primary	First Molar	Total	1928	22	1.1 ^c^	0.818 ^y^
		54	476	7	1.5	
		64	456	6	1.3	
		74	482	6	1.2	
		84	514	3	0.6	
	Second Molar	Total	2186	28	1.3 ^c^	
		55	530	10	1.9	
		65	529	8	1.5	
		75	560	5	0.9	
		85	567	5	0.9	

^x^ Pearson’s chi-square test; ^y^ Yates’ continuity correction. Values are presented as n and %. Different superscript letters (^a–c^) indicate statistically significant differences between molar types within the same dentition (*p* < 0.05).

**Table 4:** Distribution of taurodontism subtypes according to dentition and jaw localization.

Dentition	Taurodontism Type	Mandible n (%)	Maxilla n (%)	Total n (%)	*p*
Permanent	Hypotaurodont	47 (90.4) ^a^	87 (72.5) ^b^	134 (77.9)	0.018 ^x^
	Mesotaurodont	4 (7.7) ^a^	29 (24.2) ^b^	33 (19.2)	
	Hypertaurodont	1 (1.9)	4 (3.3)	5 (2.9)	
Primary	Hypotaurodont	18 (94.7)	29 (93.5)	47 (94.0)	1.000 ^y^
	Mesotaurodont	0 (0.0)	0 (0.0)	0 (0.0)	
	Hypertaurodont	1 (5.3)	2 (6.5)	3 (6.0)	
Total	Hypotaurodont	65 (91.5) ^a^	116 (76.8) ^b^	181 (81.5)	0.018 ^x^
	Mesotaurodont	4 (5.6) ^a^	29 (19.2) ^b^	33 (14.9)	
	Hypertaurodont	2 (2.8)	6 (4.0)	8 (3.6)	

^x^ Monte Carlo-corrected Fisher’s exact test; ^y^ Fisher’s exact test. Values are presented as n (%). Percentages represent the distribution of taurodontism subtypes within the affected teeth of each category. Different superscript letters (^a,b^) indicate statistically significant differences between jaws within the same dentition (*p* < 0.05).

**Table 5:** Distribution of taurodontism subtypes in affected teeth according to age group (tooth-based, n = 222).

Age Group	Hypotaurodont n (%)	Mesotaurodont n (%)	Hypertaurodont n (%)	*p*-Value
6–7 years	42 (93.3)	0 (0.0)	3 (6.7)	
8–12 years	71 (88.8)	4 (5.0)	5 (6.3)	
13–16 years	68 (70.1)	29 (29.9)	0 (0.0)	<0.001 ^a^

^a^ Monte Carlo-corrected Fisher’s exact test. Values represent the number of affected teeth (n = 222) and are presented as n (%). Statistical significance was set at *p* < 0.05.

**Table 6:** Distribution of taurodontism subtypes according to molar type.

Dentition	Taurodontism Type	1st Molar n (%)	2nd Molar n (%)	*p*-Value
Permanent	Hypertaurodont	5 (5.3) ^a^	0 (0.0) ^b^	0.009 ^x^
	Hypotaurodont	77 (81.9)	57 (73.1)	
	Mesotaurodont	12 (12.8) ^a^	21 (26.9) ^b^	
Primary	Hypertaurodont	2 (9.1)	1 (3.6)	0.576 ^y^
	Hypotaurodont	20 (90.9)	27 (96.4)	
	Mesotaurodont	0 (0.0)	0 (0.0)	

^x^ Monte Carlo-corrected Fisher’s exact test. ^y^ Fisher’s exact test. Values are presented as n (%). Different superscript letters (^a,b^) indicate statistically significant differences between the first and second molars within the same dentition (*p* < 0.05).

**Table 7:** Symmetry patterns and distribution of associated dental anomalies in patients with taurodontism (n = 77).

Characteristics	Frequency, n (%)
Symmetry Patterns	
Bilateral occurrence	66 (85.7)
Unilateral occurrence	11 (14.3)
Right side	5 (6.5)
Left side	6 (7.8)
Associated Dental Anomalies	
None (isolated taurodontism)	62 (80.5)
Presence of at least one anomaly	15 (19.5)
Root dilaceration	8 (10.4)
Hypodontia	4 (5.2)
Pyramidal molar	2 (2.6)
Pulp stone	1 (1.3)

McNemar test analysis revealed no significant difference between the right and left sides (*p* = 1.000). Values are presented as n (%).

**Table 8:** Distribution of pyramidal molars according to sex (patient-based) and dentition type (tooth-based).

Variables	Total Examined (N)	Pyramidal Present n (%)	Pyramidal Absent n (%)	*p*-Value
Sex (Patient-based)	1167	18 (1.54%)	1149 (98.46%)	0.022 ^a^
Male	605	4 (0.66%)	601 (99.34%)	
Female	562	14 (2.49%)	548 (97.51%)	
Dentition Type (Tooth-based)	9085	75 (0.83%)	9010 (99.17%)	0.025 ^b^
Permanent	4971	51 (1.03%)	4920 (98.97%)	
Primary	4114	24 (0.58%)	4090 (99.42%)	

^a^ Yates’ continuity correction. ^b^ Pearson’s chi-square test. Values are presented as n (%). Statistical significance was set at *p* < 0.05.

**Table 9:** Prevalence of pyramidal morphology according to molar type in permanent and primary dentitions (tooth-based, n = 75 affected teeth).

Dentition	Molar Type	Total Evaluated (N)	Pyramidal Present, n (%)	*p*-Value
Permanent	First molar	3419	17 (0.50%)	0.001 ^a^
	Second molar	1552	34 (2.19%)	
Primary	First molar	1928	12 (0.62%)	>0.05 ^a^
	Second molar	2186	12 (0.55%)	

^a^ Pearson’s chi-square test. Statistical significance was set at *p* < 0.05.

**Table 10:** Distribution of pyramidal molars according to jaw localization and side (tooth-based, n = 75 affected teeth).

Variables	Permanent (n = 51)	Primary (n = 24)	Total (n = 75)	*p*-Value
Jaw Localization				0.819 ^a^
Maxilla	27 (52.9%)	12 (50.0%)	39 (52.0%)	
Mandible	24 (47.1%)	12 (50.0%)	36 (48.0%)	
Side Distribution				0.733 ^a^
Right side	26 (51.0%)	12 (50.0%)	38 (50.7%)	
Left side	25 (49.0%)	12 (50.0%)	37 (49.3%)	

^a^ Pearson’s chi-square test. Values are presented as n (%). Statistical significance was set at *p* < 0.05.

**Table 11:** Symmetry characteristics of pyramidal molars (patient-based).

Characteristics	Frequency, n (%)
Bilateral occurrence	13 (72.2)
Unilateral occurrence	5 (27.8)
Total	18 (100)

McNemar test showed no significant difference between the right and left sides (*p* = 1.000). Values are presented as n (%).

## Data Availability

The data presented in this study are available from the corresponding author on reasonable request. The data are not publicly available due to ethical and privacy restrictions.

## Abbreviations

CBCT	Cone-beam computed tomography
CEJ	Cementoenamel junction
DLX2	Distal-less homeobox 2
HERS	Hertwig’s epithelial root sheath
PTCH2	Patched 2
SHH	Sonic hedgehog
